# Association between heart rate variability metrics from a smartwatch and self-reported depression and anxiety symptoms: a four-week longitudinal study

**DOI:** 10.3389/fpsyt.2024.1371946

**Published:** 2024-05-31

**Authors:** Young Tak Jo, Sang Won Lee, Sungkyu Park, Jungsun Lee

**Affiliations:** ^1^Department of Psychiatry, Kangdong Sacred Heart Hospital, Hallym University College of Medicine, Seoul, Republic of Korea; ^2^Department of Psychiatry, Kyungpook National University Chilgok Hospital, Kyungpook National University School of Medicine, Daegu, Republic of Korea; ^3^Department of Artificial Intelligence Convergence, Kangwon National University, Chuncheon, Republic of Korea; ^4^Department of Psychiatry, Asan Medical Center, University of Ulsan College of Medicine, Seoul, Republic of Korea

**Keywords:** heart rate variability (HRV), photoplethysomography (PPG), smartwatch, depression, anxiety

## Abstract

**Background:**

Elucidating the association between heart rate variability (HRV) metrics obtained through non-invasive methods and mental health symptoms could provide an accessible approach to mental health monitoring. This study explores the correlation between HRV, estimated using photoplethysmography (PPG) signals, and self-reported symptoms of depression and anxiety.

**Methods:**

A 4-week longitudinal study was conducted among 47 participants. Time–domain and frequency–domain HRV metrics were derived from PPG signals collected via smartwatches. Mental health symptoms were evaluated using the Patient Health Questionnaire-9 (PHQ-9) and Generalized Anxiety Disorder-7 (GAD-7) at baseline, week 2, and week 4.

**Results:**

Among the investigated HRV metrics, RMSSD, SDNN, SDSD, LF, and the LF/HF ratio were significantly associated with the PHQ-9 score, although the number of significant correlations was relatively small. Furthermore, only SDNN, SDSD and LF showed significant correlations with the GAD-7 score. All HRV metrics showed negative correlations with self-reported clinical symptoms.

**Conclusions:**

Our findings indicate the potential of PPG-derived HRV metrics in monitoring mental health, thereby providing a foundation for further research. Notably, parasympathetically biased HRV metrics showed weaker correlations with depression and anxiety scores. Future studies should validate these findings in clinically diagnosed patients.

## 1 Introduction

Mental health disorders constitute a major global public health concern, with an escalating number of patients requiring mental health services with considerable social cost (1). Depression and anxiety are the two most disabling mental disorders, ranked among the top 25 leading causes of healthcare burden worldwide in 2019 (2). In South Korea, the problem is severe, as the suicide rate has consistently ranked first in the OECD for more than 10 years, with around 25 people per 100,000 resorting to suicide each year (3). The disease burden of mental and behavioral disorders was estimated to account for 6.4% of the total disease burden in South Korea (4). The importance of mental health services therefore cannot be overstated.

Traditionally, patients receive diagnoses through face-to-face consultations with psychiatrists, which pave the way for various treatments such as counseling, medication, and hospitalization. However, this approach can render mental health services less accessible in certain areas or under specific circumstances (5). For instance, during the recent COVID-19 pandemic, the number of patients with depression surged; however, providing appropriate services proved challenging due to social distancing measures and other factors (6). Even outside of pandemic conditions, it is consistently reported that current mental health services are unable to cope with the rapid increase in the number of psychiatric patients (7). Furthermore, face-to-face consultations have inherent limitations; they rely on individuals’ ability to recollect their symptoms, which can introduce significant bias (8).

Given these limitations in the provision and access to adequate mental health services under the current system, applications of digital technologies are increasing (9–11). Digital technologies can help overcome the issue of accessibility in providing mental health services and can also alleviate recollection bias by offering real-time physiological digital markers to both physicians and patients. Commercialized services for some conditions, such as insomnia, already exist. For instance, the Sleep Healthy Using the Internet (SHUTi) service has been effective for insomnia and significantly reduced depression and anxiety symptoms (12). Moreover, studies on mobile intervention platforms for insomnia in South Korea have emphasized the potential of wearable devices (13). The mobile and wearable devices enable scalable sampling of the experiences and feelings of a patient (14), thus facilitating well-being reports collection systematically and objectively at scale (15). Wearable devices not only address accessibility concerns in traditional healthcare, but also enable healthcare providers to achieve more precise diagnoses through continuous collection of real-time patient biometrics, allowing physicians to analyze a patient’s condition over a broader spectrum (16).

Nevertheless, the information that wearable devices can collect is somewhat restricted in both quality and quantity compared with medical devices. Therefore, clinically relevant mental health data of the user is a priority for wearable devices. Research has subsequently expanded to heart rate variability (HRV) (17), referring to the small variations between heartbeat cycles. In a healthy human heart, a dynamic relationship exists between the parasympathetic nervous system (PNS) and the sympathetic nervous system, often referred to as autonomic nervous system balance. Consequently, HRV is associated with numerous psychiatric symptoms. It has been suggested that patients with depression exhibit lower HF power, which indicate a diminished regulatory ability of the parasympathetic nervous system and short-term flexibility of the autonomic nervous system, respectively (18). In addition, a meta-analysis has revealed that anxiety disorders are associated with significant reductions in both high-frequency and time–domain HRV metrics. This reduction may signify a failure of inhibition, characterized by a diminished capacity to inhibit responses, leading to decreased vagal outflow and lower HRV (19). Furthermore, it has been suggested that the LF/HF ratio, generally indicative of autonomic balance, may reflect aspects of an individual’s resilience profile (20).

However, HRV is traditionally measured using an electrocardiogram, which is time-consuming and resource-intensive. Therefore, various methods to measure HRV using scalable devices, such as wearables, have been developed (21, 22). Among them, the photoplethysmography (PPG) method is favored owing to its reliability compared with the gold standard electrocardiogram method (23–25). Therefore, in this study, we aimed to advance this exploration and investigate whether HRV measured using wearable devices could be applied to depression and anxiety. This study will elucidate whether real-time signals measured through wearable devices correlate with patient mental health. We therefore examined the association between HRV metrics collected in real-time while wearing a smartwatch and self-reported depression and anxiety in healthy adults.

## 2 Materials and methods

### 2.1 Study participants

The study initially recruited young adults who studied or worked at the Korean Advanced Institute of Science and Technology (KAIST) and the Institute for Basic Science. We excluded individuals with comorbid medical or psychiatric conditions; therefore, those with a formal diagnosis of depression or other psychiatric disorders were not included in the study. However, participants with a certain level of depressive or anxiety symptoms, which did not meet the diagnostic criteria for psychiatric disorders such as major depressive disorder (MDD), were still eligible. Additionally, we excluded individuals with limited access to Wi-Fi and night or shift workers to avoid bias in biomedical signal interpretation. This four-week experiment ran from March 8th to April 4th, 2021. All of the data was anonymized prior to the analysis. This study was approved by the Institutional Review Board of KAIST (KH2020–027).

### 2.2 Psychiatric symptom assessment

Participants were requested to complete online assessments of psychiatric symptoms, such as depression and anxiety. SurveyMonkey (https://www.surveymonkey.com/), an online survey platform, was utilized to formulate a questionnaire that evaluated symptoms of depression and anxiety. Participants had to complete the survey three times at a two-week interval: baseline, week 2, and week 4. For depressive symptoms, the PHQ-9 questionnaire was used (26), while the GAD-7 questionnaire (27) was used to assess anxiety symptoms. The PHQ-9 and GAD-7 are brief self-report questionnaires comprising 9 and 7 questions, respectively. Higher scores on these questionnaires suggest more severe levels of depression and anxiety.

### 2.3 Collection and processing of biomedical signals

**Figure 1:**
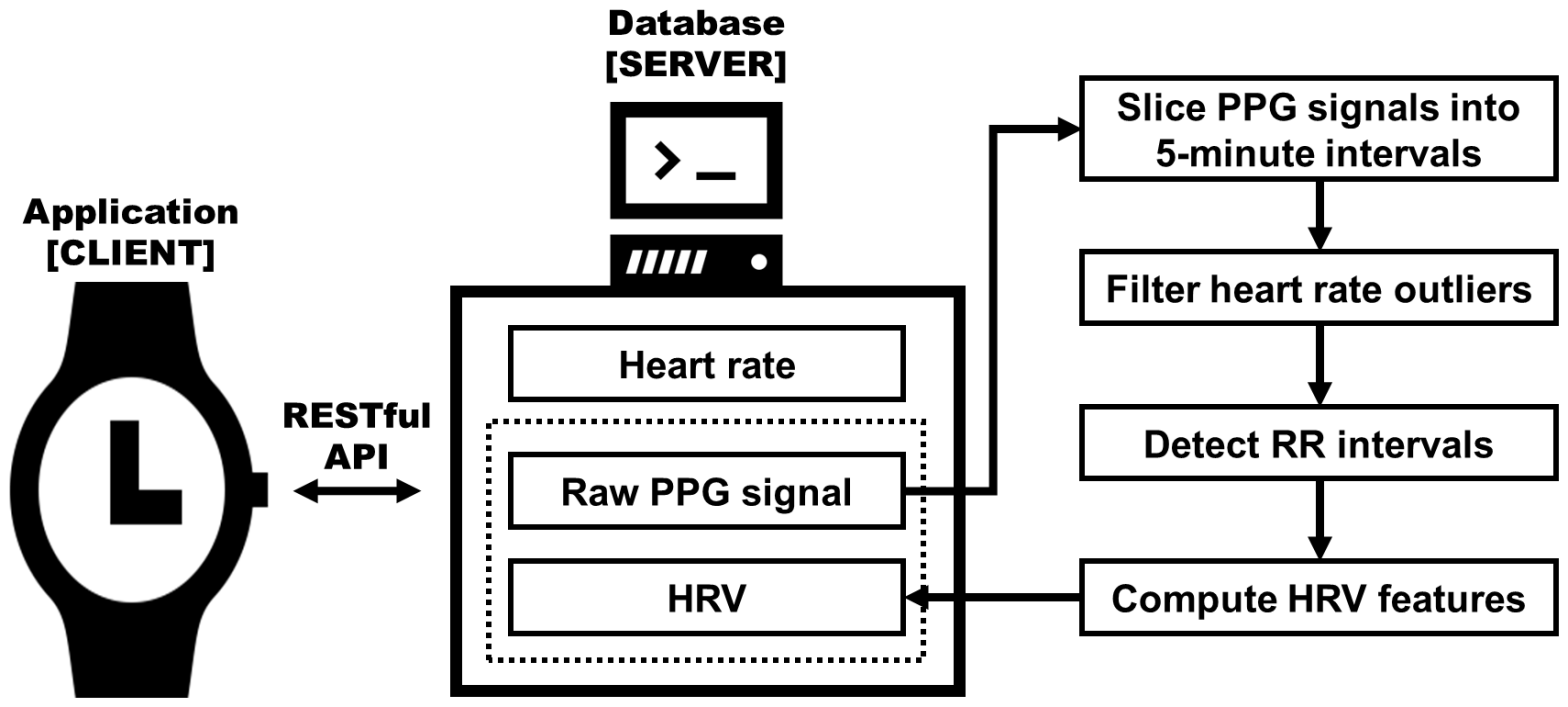
Diagram of the HRV extracting system architecture. Raw photoplethysmogram (PPG) signals extracted from a smartwatch are processed to compute heart rate variability (HRV) metrics, which are then stored in the server database; adapted from Aitolkyn et al., 2023 (30).

We utilized the Samsung Galaxy Active 2 (Samsung Electronics, Seoul, Korea) for the continuous collection of biomedical signals over a four-week period. Participants were required to wear this device at all times, enabling the uninterrupted gathering of signals. These signals were automatically uploaded to a central web server every 30 minutes, provided the device had Wi-Fi connectivity. On the server side, data were stored in a MongoDB database instance. Among the various signals gathered through wearable devices, this study predominantly focused on collecting PPG signals to measure HRV. The PPG signal was sampled every 100 ms (10 Hz), to enable continuous recordings throughout the day while ensuring battery life. The continuous PPG signal was segmented into consecutive 5-minute slices for later HRV analysis. Each slice was passed through a bandpass filter to remove frequency outliers not corresponding to human heart rates, based on the Nyquist-Shannon theorem. Subsequently, we used the HeartPy algorithm (28, 29) to identify RR intervals. From these intervals, we calculated HRV parameters for each signal slice. **Figure 1** presents the processing pipeline for conducting HRV analysis on the raw PPG signal. Further detail is described elsewhere (30, 31).

### 2.4 HRV metrics

Several metrics have been studied for HRV. This study analyzed certain time–domain and frequency–domain measures by referencing existing literature (32). Initially, we evaluated the root mean square of successive differences (RMSSD) between normal heartbeats, the standard deviation of the inter-beat interval of normal sinus beats (SDNN), the standard deviation of successive differences between normal heartbeats (SDSD), and the percentage of successive differences between normal heartbeats that differ from each other by more than 50 ms (PNN50) as time–domain measures to quantify the extent of variability. Next, we assessed the absolute power of the low-frequency band (LF), the absolute power of the high-frequency band (HF), and LF/HF ratio as frequency–domain measures after dividing HRV into different frequency bands using a Fast Fourier Transformation. **Table 1** provides a detailed description of each HRV metric.

**Table 1:** Overview of investigated HRV metrics.

Parameter	Description
Time–domain measures	
RMSSD (ms)	Root mean square of successive RR interval differences
SDNN (ms)	Standard deviation of NN intervals
SDSD (ms)	Standard deviation of successive RR interval differences
PNN50 (%)	Percentage of successive RR intervals that differ by more than 50 ms
Frequency–domain measures	
LF (ms^2^)	Absolute power of the low-frequency band (0.04–0.15 Hz)
HF (ms^2^)	Absolute power of the high-frequency band (0.15–0.4 Hz)
LF/HF	Ratio of LF-to-HF power.

### 2.5 Statistical analysis

The collected biomedical signals were analyzed at 2-week increments. The HRV metric was split into the first 2 weeks and the subsequent 2 weeks; the mean HRV metric was calculated for each period. These variables were then correlated with the self-reported depression and anxiety scores of each participant. Considering the potential delay in the temporal relationship between HRV and psychiatric symptoms, the mean HRV metrics of the first 2 weeks were correlated with clinical measures at baseline, and weeks 2 and 4, while the mean HRV metrics of the subsequent 2 weeks were correlated with clinical measures at weeks 2 and week 4. Pearson correlation analysis was applied, and a p-value of < 0.05 was considered statistically significant. Additionally, we performed sensitivity analyses for each gender to account for differences in cardiac electrophysiology (33), and psychiatric symptoms (34). All statistical analyses were conducted using R version 4.1.3 (35).

## 3 Results

### 3.1 Characteristics of study participants

A total of 47 participants were included in the study, 24 males and 23 females, with an average age of 28.7 (5.79) years. The average height of the participants was 169.0 (6.38) cm, and the average weight was 63.1 (11.5) kg. The study participants comprised 13 undergraduates, 17 graduate students, and 17 office workers. A total of 25 participants (53.2%) had previously used a personal smartwatch before the study. The mean PHQ-9 score of the study participants taken via self-report questionnaires was 3.62 (3.69) at baseline, 3.75 (3.69) at week 2, and 3.75 (3.78) at week 4. The mean GAD-7 score was 3.02 (3.53) at baseline, 2.53 (2.91) at week 2, and 2.45 (2.69) at week 4. There were no reported physiological or psychological adverse effects from wearing the watch throughout the study period. Despite some participants occasionally forgetting to wear the watch, all experiments were successfully completed with the support and continuous monitoring provided by researchers throughout the study period. The demographics and clinical characteristics of study participants are presented in **Table 2**.

**Table 2:** Demographics and clinical characteristics of study participants.

Variables	Value
Demographics	
Sex (M:F)	24:23
Age (years)	28.7 (5.79)
Height (cm)^1^	169.0 (6.38)
Weight (kg)^2^	63.1 (11.5)
Job (n)	Undergraduate 13 Graduate 17 Office-worker 17
Prior smartwatch use (n)	Yes 25 No 22
Clinical variables	
Initial PHQ-9	3.617 (3.692)
PHQ-9 at week 2	3.745 (3.686)
PHQ-9 at week 4	3.745 (3.779)
Initial GAD-7	3.021 (3.529)
GAD-7 at week 2	2.532 (2.911)
GAD-7 at week 4	2.447 (2.693)

Mean (Standard deviation); ^1^9 missing data; ^2^10 missing data.

### 3.2 Correlations between HRV and depression

Regarding time–domain measures, the mean RMSSD for the first 2 weeks did not significantly correlate with PHQ-9 scores at any time point. However, the mean RMSSD for the subsequent 2 weeks significantly correlated with the PHQ-9 score measured at week 2 (r = -0.329, p = 0.024). The mean SDNN for the first 2 weeks significantly correlated with the PHQ-9 score measured at the initial assessment (r = -0.310, p = 0.034), while the mean SDNN for the subsequent 2 weeks correlated significantly with PHQ-9 scores measured at weeks 2 and 4 assessments (r = -0.327, p = 0.025; r =3-0.301, p = 0.040, respectively). Only the mean SDSD of the subsequent 2 weeks showed a significant correlation with the PHQ-9 score of week 2 (r = -0.356, p = 0.014).

Regarding frequency–domain measures, the mean LF for the first 2 weeks significantly correlated with the PHQ-9 score at the initial assessment (r = -0.369, p = 0.011). The mean LF for the subsequent 2 weeks was significantly correlated with the PHQ-9 scores at weeks 2 and 4 (r = -0.355, p = 0.014; r = -0.341, p = 0.019, respectively). The mean LF/HF ratio of the first 2 weeks significantly correlated with the PHQ-9 score at the initial assessment (r = -0.363, p = 0.012). However, no significant correlation was found between the mean HF for any period and the measured PHQ-9 scores. The correlations between HRV metrics and depressive symptoms measured by PHQ-9 are presented in **Table 3**.

**Table 3:** Correlations between HRV metrics and depressive symptoms measured by PHQ-9 scores.

	Initial PHQ-9			PHQ-9 at week 2			PHQ-9 at week 4		
	r	stat	p	r	stat	p	r	stat	p
RMSSD-1	-0.145	-0.985	0.330	-0.157	-1.064	0.293	-0.098	-0.661	0.512
RMSSD-2	–	–	–	-0.329	-2.337	0.024*	-0.265	-1.847	0.071
SDNN-1	-0.310	-2.188	0.034*	-0.225	-1.549	0.128	-0.199	-1.364	0.179
SDNN-2	–	–	–	-0.327	-2.318	0.025*	-0.301	-2.120	0.040*
SDSD-1	-0.165	-1.124	0.267	-0.214	-1.468	0.149	-0.104	-0.701	0.487
SDSD-2	–	–	–	-0.356	-2.558	0.014*	-0.285	-1.991	0.053
PNN50–1	-0.123	-0.833	0.409	-0.080	-0.536	0.594	-0.081	-0.546	0.588
PNN50–2	–	–	–	-0.232	-1.600	0.117	-0.230	-1.583	0.121
LF-1	-0.369	-2.660	0.011*	-0.233	-1.610	0.114	-0.228	-1.573	0.123
LF-2	–	–	–	-0.355	-2.544	0.014*	-0.341	-2.429	0.019*
HF-1	-0.176	-1.197	0.238	-0.123	-0.833	0.409	-0.110	-0.741	0.462
HF-2	–	–	–	-0.270	-1.878	0.067	-0.254	-1.764	0.085
LF/HF-1	-0.363	-2.611	0.012*	-0.246	-1.700	0.096	-0.220	-1.510	0.138
LF/HF-2	–	–	–	-0.207	-1.419	0.163	-0.161	-1.096	0.279

Pearson correlation coefficient; The number after the HRV metric refers to the first or second half of the observation period, respectively. *Statistically significant p < 0.05.

### 3.3 Correlations between HRV and anxiety

For time–domain measures, the mean RMSSD and PNN50 showed no significant correlations with GAD-7 scores at any time point. The mean SDNN and SDSD showed statistically significant correlations. Specifically, the mean SDNN for the first and the subsequent 2 weeks showed significant correlations with the GAD-7 score measured at week 2 (r = -0.300, p = 0.040; r = -0.293, p = 0.046), and the mean SDSD of the first 2 weeks showed a significant correlation with the GAD-7 score of week 2 (r = -0.310, p = 0.034).

For frequency–domain measures, the mean LF for the first 2 weeks significantly correlated with the GAD-7 score measured at the initial assessment (r = -0.292, p = 0.047). The mean LF for the subsequent 2 weeks also significantly correlated with the GAD-7 score measured at week 2 (r = -0.323, p = 0.027). However, neither the mean HF nor the LF/HF ratio significantly correlated with GAD-7 scores at any time point. The correlations between HRV metrics and anxiety symptoms measured by GAD-7 are presented in **Table 4**.

**Table 4:** Correlations between HRV metrics and anxiety symptoms as measured by GAD-7 scores.

	Initial GAD-7			GAD-7 at week 2			GAD-7 at week 4		
	r	stat	p	r	stat	p	r	stat	p
RMSSD-1	-0.136	-0.919	0.363	-0.268	-1.862	0.069	-0.033	-0.219	0.828
RMSSD-2	–	–	–	-0.248	-1.714	0.093	-0.189	-1.289	0.204
SDNN-1	-0.268	-1.867	0.069	-0.300	-2.112	0.040*	-0.066	-0.442	0.661
SDNN-2	–	–	–	-0.293	-2.052	0.046*	-0.202	-1.380	0.174
SDSD-1	-0.130	-0.878	0.385	-0.310	-2.189	0.034*	-0.003	-0.019	0.985
SDSD-2	–	–	–	-0.255	-1.773	0.083	-0.169	-1.147	0.257
PNN50–1	-0.143	-0.970	0.337	-0.209	-1.433	0.159	-0.058	-0.390	0.698
PNN50–2	–	–	–	-0.234	-1.612	0.114	-0.204	-1.400	0.169
LF-1	-0.292	-2.045	0.047*	-0.249	-1.722	0.092	-0.060	-0.403	0.689
LF-2	–	–	–	-0.323	-2.291	0.027*	-0.219	-1.506	0.139
HF-1	-0.175	-1.194	0.239	-0.238	-1.647	0.107	-0.057	-0.380	0.706
HF-2	–	–	–	-0.272	-1.897	0.064	-0.212	-1.456	0.152
LF/HF-1	-0.249	-1.722	0.092	-0.101	-0.681	0.499	-0.063	-0.421	0.676
LF/HF-2	–	–	–	-0.132	-0.890	0.378	-0.066	-0.445	0.658

Pearson correlation coefficient; The number after the HRV metric refers to the first or second half of the observation period, respectively. *Statistically significant p-value < 0.05.

### 3.4 Sensitivity analyses

We performed sensitivity analyses for each gender. Overall, the results remained consistent when correlations were evaluated among males, females, or the group as a whole. However, when analyzing correlations within a single gender, the number of significant correlations between HRV metrics and clinical measures decreased. This might be attributed to an insufficient sample size, reducing statistical power. The overall trend of correlation was the same across genders, with only differences in the p-values. The sole exception was observed in male participants, where the mean SDNN for the first 2 weeks significantly correlated with the PHQ-9 score measured at the final assessment (r = -0.452, p = 0.026).

## 4 Discussion

The association between HRV and psychiatric symptoms is acknowledged in the literature (18–20, 36). From a physiological perspective, the abnormal serotonergic system observed in various psychiatric conditions may contribute to cardiovascular dysregulation through alterations in endocrine and autonomic functions (37). This relationship is thought to be partly modulated by the hypothalamic-pituitary-adrenal (HPA) axis. Furthermore, because the serotonin transporter is predominantly found in platelets – where serotonin acts as a vasoconstrictor – a potential pathogenic link between psychiatric conditions and cardiovascular dysregulations may exist (38).

In line with previous literature, we found that some HRV metrics were significantly correlated with clinical measures with self-report questionnaires. What distinctly sets our findings apart from previous studies is that we obtained HRV data from PPG signals collected via a highly accessible wearable device, suggesting its potential to monitor mental health across a wide demographic range. Furthermore, we observed somewhat different patterns of associations between HRV metrics and clinical measures. Previous studies have suggested that HF is significantly associated with depression or anxiety, as lower HF power is linked to stress, panic, or anxiety (39). The relationships between time–domain measures and clinical measures are complex, yet multiple correlations have been continuously reported (18, 19). In contrast, our study results demonstrated correlations between LF and depression or anxiety, rather than HF. Furthermore, the LF/HF ratio showed only one significant correlation coefficient with clinical measures. Moreover, only few correlations were observed between time–domain measures, except for SDNN, and depression or anxiety.

Nevertheless, our results are partially consistent with cardiac electrophysiology. The LF band (0.04–0.15Hz), which predominantly reflects baroreceptor activity under resting conditions (40), may plausibly be associated with mental health conditions. For the LF/HF ratio, while it is often thought to reflect the balance of sympathetic and parasympathetic nervous systems, it has also been suggested that this ratio does not always reflect autonomic balance (41). Regarding time–domain measures, SDNN, generally considered the gold standard for clinical HRV metrics and medical stratification of cardiac risk (42), showed more than one correlations with clinical measures. A Similar HRV metric, SDSD, also showed two significant correlations with clinical measures. On the other hand, the RMSSD, a measure of beat-to-beat variance in heart rate representing vagal-mediated changes reflected in HRV and related to the parasympathetic activity (43), showed only one significant correlation. Similarly, PNN50, although recognized as a less sensitive measure of the PNS, was not significantly correlated with clinical measures in this study. These results suggested that HRV is related to the balance of sympathetic and parasympathetic nervous systems; however, metrics biased toward the parasympathetic region are less likely to be associated with depression and anxiety.

Among the significant associations between HRV metrics and clinical measures, all metrics from the first two weeks were associated only with clinical measures at baseline or week 2. Similarly, HRV metrics from the following two weeks were associated with clinical measures at weeks 2 or 4. This suggests that HRV metrics may reflect temporal changes in psychiatric symptoms. However, the inconsistency in statistical significance across items, the relatively small number of overall participants and their measured PPG signals, and the division of the entire observation period into two halves create difficulty in comprehensively interpreting the temporal association between HRV and clinical symptoms based solely on the results of this study.

This study had several limitations. First, we recruited participants from specific contexts, limiting the generalizability of our results. Our study population consisted of well-educated young adults, not a diverse demographic. This limits the scalability of our findings, as groups such as the elderly – who have less access to digital technologies – might display different characteristics. Moreover, extending our results to children or adolescents should be done with caution, as their cardiac electrophysiology differs from that of adults (44). Second, most participants were asymptomatic or exhibited mild symptoms, as indicated by mean scores around three on the PHQ-9 and GAD-7. This necessitates cautious statistical interpretation of the correlations due to potential bias. A separate study with a different population is required to confirm clinical utility. Third, all symptom assessments were self-reported, which risks social desirability response bias (45) and may not accurately reflect true symptoms. Furthermore, since the survey was web-based, we cannot confirm that the actual respondents were the intended participants. Meta-analyses have shown that clinician-rated depressive symptoms have a significantly larger effect size than self-reported symptoms (46). Incorporating clinician interviews or clinician-rated scales could yield more comprehensive results. Fourth, although we excluded participants diagnosed with any psychiatric comorbidity, including alcohol and other substance use disorders, we could not rule out the use of substances like caffeine or alcohol during the actual experiment period. Nevertheless, considering our study’s aim to evaluate the utility of a smartwatch as a real-time monitor for HRV as a digital biomarker of psychiatric symptoms, the consumption of certain substances within daily intake ranges should be considered part of our research. Lastly, the quality of biomedical signals collected by smartwatches has been a subject of ongoing debate. Specifically, PPG signals are argued to not accurately represent HRV, especially under free-living conditions (47) or without controls for breathing (48). Nevertheless, we did filter out noise from the PPG signals throughout the processing pipeline (31). This study has demonstrated that estimated HRV from PPG signals is significantly correlated with ECG-measured HRV metrics, indicating the reliability of our estimated HRV.

Despite these limitations, our study is significant because it demonstrated that biomedical signals obtained through simple methods, such as common smartwatches, are associated with psychiatric symptoms such as depression and anxiety. Further research should aim to highlight the existing shortage of mental health services. Additionally, with the integration of digital technology, these methods can provide more advanced mental health services in conjunction with the digital platforms that have gained substantial attention. We look forward to future research on a broader range of mental health symptoms based on various biomedical signals, not just PPGs, and research on a wider patient population.

## Data availability statement

The datasets presented in this article are not readily available because of local ethical restrictions. Requests to access the datasets should be directed to shaun@kangwon.ac.kr.

## Ethics statement

The studies involving humans were approved by Institutional Review Board of KAIST (KH2020-027). The studies were conducted in accordance with the local legislation and institutional requirements. The participants provided their written informed consent to participate in this study.

## Author contributions

YJ: Writing – original draft, Validation, Formal analysis, Conceptualization. SL: Writing – review & editing, Methodology, Investigation, Funding acquisition, Conceptualization. SP: Writing – review & editing, Software, Methodology, Investigation, Data curation, Conceptualization. JL: Writing – review & editing, Methodology, Funding acquisition, Formal analysis, Conceptualization.
